# The lived experience and supportive care needs of Australian parents caring for children with Epidermolysis Bullosa: a qualitative descriptive analysis

**DOI:** 10.1186/s13023-025-04159-4

**Published:** 2025-11-29

**Authors:** Hayley Ruf, Colin J. Ireland, Lemuel J. Pelentsov, Zlatko Kopecki

**Affiliations:** 1 Clinical and Health Sciences, University of South Australia, Adelaide, Australia; 2 Future Industries Institute, University of South Australia, Adelaide, Australia; 3 Dystrophic Epidermolysis Bullosa Research Association (DEBRA) International Am Heumarkt, Austria and DEBRA Australia, Pittsworth, Australia

**Keywords:** Epidermolysis Bullosa, Supportive care needs, Parents

## Abstract

**Background:**

Inherited epidermolysis bullosa (EB) is a rare, incurable genodermatosis characterised with recurrent skin blistering and mucosal fragility. Wound care and nursing are critical to everyday lives of children living with EB, while the profound effect of caring for a child with a painful genetic condition leaves a significant effect on quality of life of parents and the family. This study aimed to better understand the lived experiences and supportive care needs of parents of children with EB in Australia.

**Methods:**

Interpretative phenomenological analysis was used, and semi structured focus groups interviews were conducted with Australian parents of children with EB. This included 18 parents of children with EB simplex (EBS), 9 parents of children with Junctional EB (JEB) and 16 parents of children with Dystrophic EB (DEB). The data were thematically analysed, all participants are residents of Australia and therefore reflect the services in this geographical location.

**Results:**

Six overarching themes included: practical needs, emotional needs, informational needs, psychological needs, social needs and physical needs.

**Conclusions:**

Parents caring for a child with EB in Australia face numerous needs and challenges regardless of EB severity which highlights the need for provision of better more comprehensive services to deliver family focused holistic EB care that can alleviate some of the burden of EB and improve the quality of life for families and individuals living with EB.

## Background

Inherited epidermolysis bullosa (EB) comprises a complex group of genetic disorders that manifest with mechanically induced fragility and blistering of skin and mucous membranes with variable clinical severity [1]. The latest consensus reclassification of inherited EB and other disorders with skin fragility described the four major classical types of EB based on level of skin cleavage at dermal-epidermal junction [2]. Epidermolysis Bullosa simplex (EBS), accounts for 40–70% of cases and is caused by skin cleavage at the basal epidermal layer, exhibiting the greatest genetic and clinical heterogeneity [3]. Dystrophic EB (DEB) is the second most common type, comprising 25–50% of cases, and results from genetic defects in type VII collagen (C7), leading to skin separation in the upper dermis. Junctional EB (JEB) is rare, occurring in 5–20% of cases, and is caused by mutations affecting hemidesmosomes, focal adhesions, or basement membrane laminin 332, often leading to early childhood mortality [4]. Kindler EB is considered an ultra-rare form, affecting fewer than 1% of cases world-wide, with only a few hundred known individuals; none were included in this study. These four main types are further divided into over 30 clinical subtypes based on mutated genes and targeted protein, with severity ranging from mild, localised involvement with minimal impact to severe, forms including multiple organs, severe multisystem disease that significantly impacts quality of life and life expectancy [5].

**Fig. 1:**
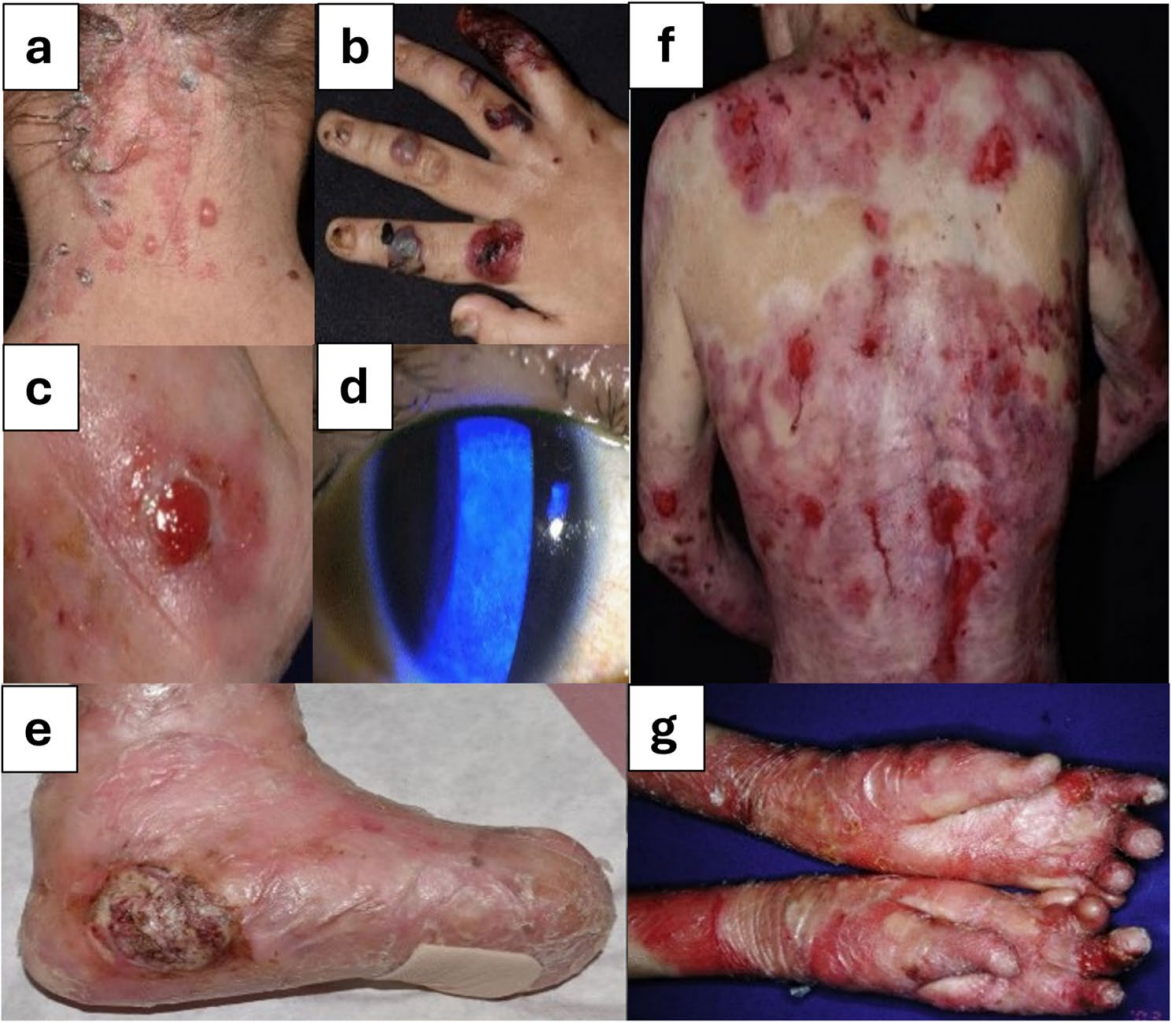
Specific features associated with main EB types. Herpetiform blistering (**a**) and acral haemorrhagic blistering and nail dystrophy (**b**) in EBS. Granulation tissue (**c**) and corneal erosions (**d**) in JEB. Squamous cell carcinoma (**e**), generalised blistering erosions and fibrosis (**f**) and pseudosyndactyly in RDEB. Adapted with permission [7]. Copyright 2021, *Am J Clin Dermatol*

Management of EB is challenging due to its rarity, complexity, and numerous extracutaneous manifestations, all of which impact the daily lives of children with EB and their families. In severe forms, disordered physiological processes lead to a cycle of delayed wound healing, abnormal skin remodelling, and increased susceptibility to serious complications. These include deformities, life-threatening infections such as sepsis, squamous cell carcinoma, malnutrition, cardiomyopathy, renal disease, and osteoporosis [6]. Figure 1 illustrates the main clinical manifestations commonly associated with the major EB subtypes [7], highlighting the extent of disease severity, systemic effects, and the significant care needs and quality-of-life impact for individuals living with EB.

Research on EB to date have primarily focused on the needs of affected children using both qualitative and quantitative approaches to assess the quality of life, including global cross-sectional surveys of patient-reported outcomes [8, 9]. Other studies have explored the burden of disease of living with severe forms of EB [8], identified helpful practices to alleviate this burden [10], and examined the needs of healthcare professionals working with children with EB and their families [11]. Over a decade ago, the EB family/parental burden score was developed and validated to assess disability based on psychological, social, physical, and economic factors. However, both this burden of disease (BOD) score and Quality of Life Questionnaire for EB (QOLEB) are highly dependent on EB subtype and disease severity, requiring adaptation and validation for specific countries and cultural contexts [12–14].

Dystrophic Epidermolysis Bullosa Research Association (DEBRA) International, a global patient advocacy and support organisation, has published psychosocial recommendations highlighting the profound impact of EB on affected children and their families. These recommendations also identify a critical research gap in understanding parental support needs as primary caregivers and how the burden of disease evolves over a child’s lifespan, influencing the entire family unit [15]. In recent years, research has increasingly focused on the lived experiences of parent’s and sibling’s in families living with EB, exploring the relationship between emotional and copping strategies, and disease management in both the short- and long-term [16, 17]. A recent study from Norway underscored the importance of healthcare professionals recognising the significant burden of extensive home care for parents of children with EB children. This aspect of caregiving is often underrecognized yet essential for providing holistic care to both the child and the family [18].

**Fig. 2:**
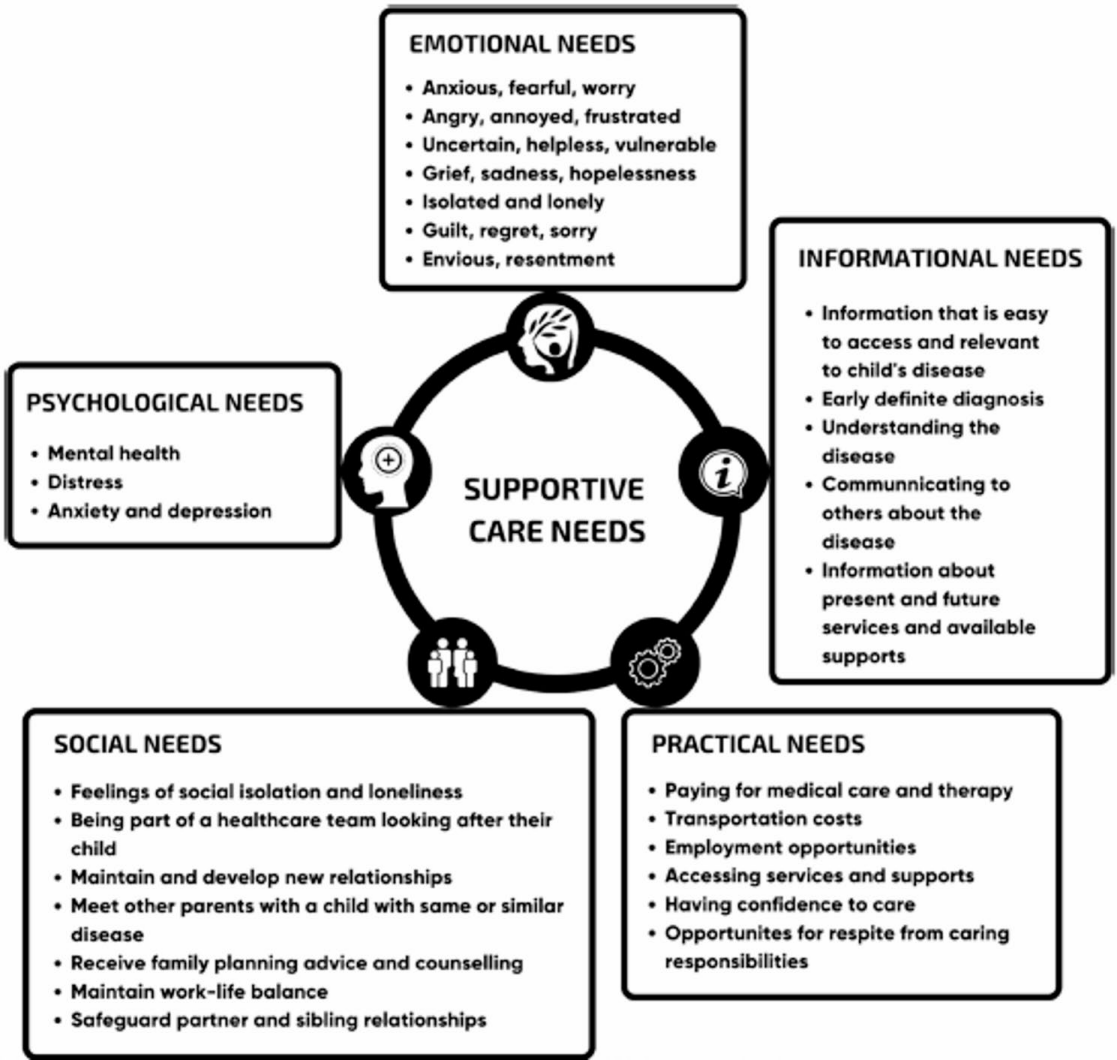
Parental supportive care needs framework [20]

The Parental Needs Scale for Rare Diseases (PNS-RD), developed by Pelentsov and colleagues [19], provides a structured framework for evaluating parental supportive care needs and was adopted for use in this study. The model comprises five key domains: emotional, informational, practical, social and psychological (Fig. 2). Further details of the development of the PNS-RD can be found elsewhere [19]. Originally designed for broad application across various rare diseases, the PNS-RD has been used in studies such as Somanadhan et al. (2025) [21], which explored the support needs of parents in Ireland. It has also been used in research focusing on specific rare diseases, including rare bleeding disorders [22] and Usher syndrome [23]. Additionally, a scoping review of literature on the support needs of parents caring for a child with EB using these domains was recently published, a sixth domain of physical was also introduced based on the EB literature [24].

DEBRA organisations worldwide have played a crucial part in supporting families living with EB, complementing existing government, non-government and healthcare systems. However, significant disparities exist in the level of support provided to parents, including access to adequate nursing care, medical supplies, psychology support, respite programs and family camps. These disparities are evident both internationally and within individual countries, such as Australia, where local/metropolitan and rural areas offer differing levels of care and support to children with EB and their families [1, 25, 26]. Few studies have examined the supportive care needs of parents caring for a child with EB and the broader impact on the family unit. There is limited evidence to imply detailing the practical and emotional burden on parents, as well as potential unmet medical needs in EB caregiving [23]. This study aims to better understand the lived experience and supportive care needs of parents caring for children with EB in Australia. The findings will contribute to the development novel support frameworks to guide healthcare professionals in delivering patient-centred care that supports individuals with EB and their families.

## Methods

### Aim

The aim of the current study was to gain a deeper understanding of the lived experiences and supportive care needs of parents caring for children with Epidermolysis Bullosa (EB) in Australia.

### Study design

This study employed a qualitative descriptive approach, as described by Sandelowski [27]. While all qualitative research involves some level of interpretation, qualitative description emphasises a lesser degree of inference compared to other methodologies, such as phenomenological or grounded theory, making it particularly suitable for an area where limited prior studies exist [27]. Focus groups were selected as the primary method of data collection, as they allowed for a broader exploration of parental experiences, encouraging discussion among participants, and facilitate the open sharing of diverse perspectives, build on peer responses, reflect on commonalities and differences, and articulate both shared and unique aspects of their experiences alike [2, 28].

### Sampling and recruitment

Parents were eligible for inclusion if they were the parent of a child diagnosed with EB. Parent recruitment was facilitated through DEBRA Australia, a not-for-profit organisation dedicated to improving the lives of children and families living with EB. Participants included parents of children with varying EB severities and subtypes, including EB Simplex (EBS), Junctional EB (JEB) and Dystrophic (DEB). Parents self-reported their child’s EB subtype and the disease severity through the discussions. While parent’s experiences may have differed in terms of required support levels, they shared common challenges and support needs, including the diagnostic journey and broader caregiving burden and responsibilities. The initial focus group was conducted by LP and ZK in Adelaide, South Australia following the EB clinic at the Women’s and Children’s Hospital. However, the COVID-19 pandemic delayed further data collection until 2022, when the opportunity presented to conduct further focus groups at the DEBRA Australia Family Camp in Sydney, New South Whales. At the family camp, ZK provided a presentation to attendees outlining the purpose of the study and invitation to parents to participate in one of three focus groups, which were then organised based on EB subtypes (EBS, JEB and DEB). Written informed consent was obtained from all parents prior to the commencement of the focus groups.

### Data collection

A total of four focus groups were conducted: three in Sydney and one in Adelaide, between August-2019 and May-2022. These interview sessions included 43 parents of children with EB, with each focus group lasting approximately one hour. Group sizes varied, ranging from nine to 18 parents. The largest group, comprising 18 parents, was conducted during a national EB family conference. Although this exceeded the conventional size for focus groups [29], it was considered advantageous as many of the parents were already familiar with one another, which fostered an environment of openness and willingness to share experiences with the researcher. A discussion guide was created with core questions derived from the PNS-RD domains of supportive care needs. However, parents were encouraged to speak freely and raise any issues they considered most significant in relation to caring for their child with EB. Discussions covered topics including parental caregiving experiences, the level of support received from family and external providers, residential location, financial impacts, and the perceived mental health effects on both parent and child. Parents were also asked to identify their greatest needs and whether they felt they received adequate support for their child. Additionally, parents were verbally presented with a summary of the main domains of supportive care needs, along with the additional domain identified through the authors scoping review. This was done to enable parents to validate or comment on the content presented, and to ensure is ongoing relevance given the extended period over which the focus groups were undertaken. The timing of several sessions to coincide with the annual EB national conference was considered particularly valuable, as it provided an opportunity to capture a wide range of perspectives in a setting where parents were already engaged and motivated to contribute.

### Ethical considerations

Ethical approval for this study was obtained from the University of South Australia Human Research Ethics Committee (#201982). Written informed consent was obtained from parents prior to their inclusion in the study.

### Data analysis

Focus group discussions were recorded and transcribed verbatim by an external service. To ensure accuracy, both the written transcripts and recordings were compared. Initial analysis was undertaken by two authors (HR and CI), who reviewed the transcripts in detail to determine whether the content aligned with one or more of the supportive care needs domains outlined in the PNS-RD (emotional, informational, practical, social, and psychological). Through an iterative process of reading the transcripts, re-listening to recordings, and extracting key participant quotes, HR and CI identified both issues that mapped directly to these domains, as well as issues that represented themes outside of the established framework. These significant quotes were then coded and compiled into a working document to facilitate deeper thematic analysis. Within this process, sub-themes were identified under each of the key domains, providing greater granularity to the data. Themes and categories were reviewed collaboratively by CI and HR to ensure consistency of interpretation. Where differences in coding and categorisation occurred, these were resolved through robust discussion until consensus was reached. In cases where quotes or issues were relevant to more than one domain, they were coded across multiple themes or sub-themes to preserve the complexity of parental experiences. The most prevalent themes were then identified, with quotes selected to demonstrate the gaps within these themes. These were then reviewed by LP and ZK for collective agreeance.

## Results & findings

### Theme 1: practical needs

Practical needs were the most frequently discussed by parents and were identified as either facilitating or hindering their ability to care effectively for their child. Interestingly, many of the parents, especially in EBS focus group, had EB themselves, which may have affected their practical needs and ability to care for their child.

### Challenges in diagnosis and accessing services

Parents most discussed practical aspects around their experiences accessing services to obtain a diagnosis. Some parents who had an EB diagnosis themselves recounted having limited information or support during their own diagnosis journey, and this lack of clarity and timely diagnosis often extended to their child. Additionally, it was also reported by parents that health care professionals often lacked an understand of EB, which contributed to delays in diagnosis.

*I was in my 30s when I got diagnosed. …it was years later that I was just at the dermatologist for skin cancers and she said*,* “I think you have this*,*’ then there was nothing again until I had my son and we were up at the hospital for something completely different and I talk about*,* “just don’t put the Band-Aids on*,*” and they told me about the clinics but I didn’t even hear anything. I didn’t even know it was something really. [Mother*,* Child EBS]*

*And the just not knowing*,* so we waited two years for a diagnosis*,* so we didn’t know how severe and what type. [Mother*,* child with EBS]*

Health Care Practitioner knowledge and understanding of care was also discussed in the context of not understanding the problems associated with EB and consequently families not getting the appropriate support needed.

*We were handed over to them […….] as a low maintenance family*,* don’t need much support….” “Low maintenance because you seemed that you had everything going –“ “We do it ourselves*,* yeah exactly. [Mother*,* child with RDEB]*

The second aspect of accessing services was around **appropriate timely services for assistance with managing EB**, this included what was promised vs. reality of receiving services which align with their child’s requirements.

*That [help] came as domestic support*,* so two hours of cleaning a fortnight that was. So*,* it didn’t actually cover anything that we needed [Mother*,* child with RDEB]*.

*…. when she turned five the district nurses stopped because they don’t do those kinds of hours after school. [Mother*,* child with RDEB]*

There did appear to be differences between states and territories within Australia and between regional and metropolitan areas.

*….having that access that you can get in Sydney and Melbourne*,* but in Queensland would be great and I’m sure other states would probably feel the same. [Mother*,* child with JEB]*

*We just went home and Googled stuff and freaked out…. So it was the not knowing and we live rurally*,* and having no one accessible. [Mother*,* child with JEB]*

The **complexity of wound care**, dressings and skills was identified as a concern for parents due to the lack of healthcare provider knowledge, but also the lack of and **expenses of suitable dressings**.

*[child] is only five and we’re trying to drum into her to say*,* “make sure you tell them that your bandage is not [okay]*,*” but it shouldn’t be up to a five-year-old. [Father*,* child with EBS]*

Parents identified the dressing schemes that are available assist significantly with the financial burden and without this support there would be significant financial burden.

*The dressing scheme*,* that really helps ease the financial burden. Once I was told about how much each of the things cost and then comparing that with*,* “what is our income?” yikes*,* if we had to fund ourselves*,* we’d be in serious trouble and that would cause such a massive impact on our family. [Mother*,* child with RDEB]*

### Theme 2: emotional needs

Emotional needs were the second most prevalent concern outlined by parents, with **frustration**,** stress and guilt being the most dominant sub-domains**. Many parents expressed difficulties managing the condition, leading to frustration. This was through advocating for their children, or feeling like they were not heard or taken seriously.

*We have to advocate on behalf of our kids all the time. We’ve got to be their eyes*,* their ears*,* and their voices. It’s so bloody hard. [Mother with EBS & child with EBS]*

Parents also reported that they were seen as the experts in their child’s condition, leading to further frustration as they were seeking for help.

*“The parents are the experts.” I don’t want to be an expert in EB. I never asked to be an expert in EB. [Father*,* child with JEB]*

Parents also expressed frustration where they were promised services, however, these were not delivered.

*We saw social workers and they promised lots of stuff but gave us nothing. [Mother*,* child with RDEB]*

Stress and guilt were also reported by parents, which stemmed from many aspects including **guilt of not being able to care for their child with EB effectively**, or, that they felt they could not give their other children without EB the same level of attention and care.

.. *is siblings*,* the burden on parents with having another child there and just getting pushed aside for all these procedures that take place in the home. That’s a real burden on us that the other child gets pushed aside [Mother*,* child with RDEB]*

*It’s very emotionally draining and you’re grieving for the child that you thought you were having. [Mother*,* child with JEB]*

### Theme 3: informational needs

Parents identified a clear requirement related to treatments and expertise for their child. Several parents identified a range of issues they were faced with, including lack of support from health care professionals, through to struggling to receive a diagnosis due to lack of healthcare worker knowledge. Parents also identified the **lack of support from GPs due to the lack of knowledge**.

*You’d go to the doctor*,* and they’d treat you like you’re a bad mum who’s not looking after your kid. … this is what we need is GPs to be educated in the simplex version that it’s not the mum who’s creating these problems*,* it is a genetic [condition]– [Mother*,* child with EBS]*.

Parents also discussed a **lack of knowledge of third-party providers** related to caring for their school-aged children.

*The school*,* no*,* they know everything*,* and she came home and she’s got bandages like they give you under surgery and the screaming to get them off was awful. [Mother*,* child with RDEB]*

Another aspect that parents mentioned related to reproductive and family planning. Parents reported concerns about having additional children, and the potential risks associated with having children and whether they would carry the EB gene.

*I could have another child with EB*,*” but I just couldn’t go another nine months without knowing.” … So*,* she’s really keen on going down that path and that’s more supported needed and genetics and probably financial support. [Parents*,* child with EBS]*

*On one hand*,* my husband has got it so we always knew there’s a chance our kid would have it but we never actually really thought about what that meant because he never really talked about it much either. [Mother*,* child with EBS]*

This was also pertinent for the children with EB and something that parents need to consider. When to bring up the topic is also important.

*[Child] actually brought that up last night. I think seeing people that have a lot more severe wounds then he does he*,* we were just watching TV and he goes “what happens when I want to have kids?” and I’m like*,* “well*,*” he’s only 14 and he picked that up and I said*,* “Well*,* you may be able to speak to the partner. You know you carry the defective gene*,* so a 50–50 shot of passing that on. [Mother*,* child with EBS]*

While not commonly discussed in each focus group there were also concerns about having information and planning including palliative care from day one. There was an **underlying fear of the unknown as the disease progressed.**

*I think it’s really important for palliative care to actually be part of the multidisciplinary team from day one. Even though that child might live till they’re 24 there are steps along the way that I’ve noticed*,* and I myself as a parent could relate to this*,* everybody’s scared and worried about talking about the dying process and mortality … they’re all worried*,* they’re all scared*,* they want to know how its going to unfold*,* what are the signs to look for*,* but no one talks about it. [Mother*,* Child with RDEB]*

### Theme 4: psychological needs

In the initial scoping review [23] psychological needs sub-domains included **acceptance**,** depression**,** anxiety**,** coping and mental health and wellness**. Within the focus groups the most discussed sub-domain was coping, followed by mental health and wellness. Many parents identifying a lack of support which led to difficulty coping. Most parents discussed the need for being able to cope and what they had to do to care for their child effectively.

*we were thrown in the deep end*,* we had no idea what we were in for. [Mother*,* child with RDEB]*

*You’re like a zombie*,* you’re exhausted*,* you’re getting up for night feeds*,* you’re trying to cope with this condition of what you might be faced with. [Mother*,* child with RDEB]*

There appeared to be a cascading effect encompassing coping skills, which led to mental health concerns stemming from a lack of support.

*…. definitely depression due to the lack of support. …. I still struggle a lot. I think if it’s not for the EB mums weekend away I don’t think I’d cope. [Mother*,* child with EBS]*

*Why us? Why him? Why? Why? Why?” and then the panic sets in and then it’s like*,* “We’ve got to deal with this for the rest of his life. How do we do this? What do we do? [Mother*,* child with JEB]*

*’You’ve got to get us out of here so that we can have some normality in our life’*,* and it was never normal again [Mother*,* child with RDEB]*

Parents did mention an **increase in online support over the last three years**,** which provided an opportunity to connect with others experiencing similar circumstances** and having the ability to ask for recommendations and strategies, they saw this as a way of being able to get advice and information from parents who had been through similar circumstances to allow them to cope better.

*that connection is getting better now because of Facebook groups*,* there’s one for women*,* there’s one for teenagers*,* there’s one for Australia*,* there’s all these different groups that have recently - in the last two or three years - have come up and that support system is really good because you can be like*,* “Okay*,* I’m having trouble with X*,* do you guys have any recommendations that work for you? [Mother*,* child with RDEB]*

### Theme 5: social needs

The social domain was not discussed frequently, and no sub-domain was significantly more prevalent. Parents expressed social needs relating to **family support**,** healthcare practitioner (HCP) knowledge and understanding and societal expectations equally**.

Issues with relationships ultimately impacted on individuals’ quality of life and psychological needs in addition to social needs.

*[I] lost all my friends*,* lost all my family*,* lost my job*,* didn’t have support there. Just was all alone. [Mother*,* child with EBS]*

Societal expectations or understanding was discussed as a concern, with friends not understanding the requirements of having a child with EB which led to social isolation.

*Heat*,* if you were supposed to go out in the heat to friends’*,* you can’t and your back really hurts – well*,* it gets hard and people don’t ask after that. [Mother*,* child with RDEB]*

*You end up not getting invited and you lose contact with those friends you had and that sort of thing*,* so social isolation for parents I think is when things are at their worst. [Mother*,* child with JEB]*

### Theme 6: physical needs

Physical needs were the least discussed, however, **respite and recovery** was a key aspect identified within the focus groups. Respite and recovery is an aspect that was noted to be difficult to achieve, with several parents receiving additional support to assist in managing their child’s condition, however not actually providing them any respite.

*It was another set of hands but it didn’t relieve me from any duties.” and “it never gave us respite [Mother*,* child with RDEB]*

Parents reported caring for their child to be physically and mentally exhausting. Physical exhaustion was associated with a lack of sleep and trying to cope with the challenging condition.

*You’re like a zombie*,* you’re exhausted*,* you’re getting up for night feeds*,* you’re trying to cope with this condition of what you might be faced with. [Mother*,* child with RDEB]*

Parents also reported the mental fatigue of advocating for their children to receive the appropriate care, with many having several different experiences with schools, some being willing to receive education, and others declining education and not listening to parents concerns regarding the needs of their child.

*We have to advocate on behalf of our kids all the time. We’ve got to be their eyes*,* their ears and their voices. [Parents*,* child with EBS]*

Parents also reported caregiving being a challenging factor, both caring for their child with EB as well as their other child who do not have the disease. Parents felt their **parental role was taken away from them**, no longer able to be the parent, they felt they are solely there to care for the child’s health needs.

*You become a nurse” … “You’re not a parent anymore really. [Mother*,* child with RDEB]*

## Discussion

This study aimed to explore the needs of parents of children with EB to assess whether their needs are being met and to identify areas for improvement in future healthcare practices. Using a qualitative approach, focus groups were conducted to explore potential gaps in parental experiences in caring for their child with EB. These findings were compared with those of the authors previous scoping review, which explored what is currently known of parental supportive care needs across domains of need – emotional, informational, physical, practical, social and psychological, and provided a global perspective on the impacts and gaps in care leading to the current study [23]. These comparisons have used primary articles from the scoping review, as well as more current literature to guide the discussion.

The focus groups identified practical needs as the most prevalent gap in care, with two primary concerns: difficulties in accessing diagnostic services; and challenges in obtaining appropriate treatment. These findings align with existing literature [30–32]. Parents emphasised the complexity of wound care, which often requires specific, individualised regimens that can take several hours per day, sometimes multiple times daily [33]. Additionally, parents highlighted a lack of healthcare provider knowledge regarding EB management, further complicating care. Has et al. [3] detailed the intricacies of wound care and the expertise required by both parents and healthcare professions to ensure appropriate treatment.

Emotional needs emerged as the second most discussed domain of need. Parents of children with different EB sub-types reported similar emotional challenges, regardless of disease severity. Frustration was a dominant theme, largely influenced by unmet practical needs related to diagnosis, treatment and ongoing management. These findings reinforce the well-established link between practical and psychological stressors [15]. Bruckner [33] similarly identified that quality of life is significantly impacted when daily care routines are burdensome, leading to increased distress and physical and emotional exhaustion. Additionally, a recent interview study on the burden of EB highlighted the emotional support as a critical in every aspect of individuals psychosocial wellbeing and daily lives of families [34].

Parents of EB children also reported stress and guilt related to their perceived impacts of EB on their other children. Siblings often feel overlooked, as the primary focus of parents remain on the child with EB [17]. Recent consensus-based guidelines on palliative and end-of-life care for individuals with EB emphasise the importance of mental health support for parents, caregivers and providers, recommending a family-centred approach that prioritises emotional and psychological well-being throughout the disease trajectory [35].

Parents in this study highlighted informational gaps in several key areas, including available treatments, expert resources, reproductive and family planning, and end-of-life care. A significant concern was the lack of guidance on family planning, including genetic testing and reproductive options. A study by Bruckner [33] reported that 58.7% of parents in their study chose not to have more children due to the emotional and practical burden of EB. Saad [36] documented two cases where targeted antenatal support enabled safer deliveries, reducing EB-related complications for both mother and newborn, underscoring the need for specialised education programs to equip maternal and paediatric healthcare providers with EB-specific knowledge and resources.

Another critical issue reported by parents in this study was the lack of structured guidance for different disease stages, including palliative care. Parents expressed a desire for early-stage discussions on disease progression, including grief management. Recent consensus-based guidelines highlight that grief should not be reserved solely for end-of-life scenarios but should be addressed throughout the disease course, recognising the physical, emotional, spiritual and cognitive losses experienced by families at various stages. This reinforces the importance of comprehensive informational and emotional support to help families navigate EB-related trauma, anticipated disease progression and improve overall quality of life [35].

Although social needs were the least discussed in focus groups, this domain revealed important new insights regarding healthcare professional knowledge and support. While the previous scoping review highlighted the importance of healthcare professional’s understanding parents’ social support needs, focus groups emphasised a related but distinct issue, specifically the impact of healthcare professional knowledge gaps on parents’ information needs.

Parents also reported social isolation, with friendships deteriorating over time due to the time-intensive care required for their child with EB, and the lack of understanding from peers consequently resulted in parents being excluded from social events further intensified perceived feelings of social isolation and disconnectedness. Bruckner [33] similarly found that EB negatively affects social relationships, though their study focused primarily on patient experiences rather than parental perspectives. These findings suggest that social support interventions are needed for both parents and affected children to mitigate isolation and enhance social inclusion.

### Limitations

This study was conducted using focus groups. Due to COVID-19 restrictions between 2020 and 2022, there were delays in data collection, which may have influenced participants’ experiences. However, given the chronic nature of EB, we do not believe these delays significantly affected the study’s overall findings and the representative groups captured a good mix of responses from parents with EB children across different geographical areas of Australia and different main EB subtype groups. However, we acknowledge that the homogenous nature of the study sample may impact the generalisability if findings. As recruitment of participants was through a single channel only, DEBRA organisation, this may have resulted in an overemphasis in the results on the role or benefits of such groups, possibly limiting the generalisability of findings to those less connected to support services. This is a common issue and has previously been discussed in the support group and rare disease literature [37, 38]. Finally, as the study was conducted in Australia, certain findings may be influenced by context-specific structures, limiting generalisability to other regions.

## Conclusion

Caring for a child with EB poses substantial burdens on both mothers and fathers, with siblings also affected in some family dynamics. This study used the validated PNS-RD to explore and further understand the supportive care needs of parents caring for a child with EB across the five domains: Practical, Emotional, Informational, Psychological, and Social needs. A sixth domain, Physical, was also included following the scoping review undertaken by the authors. Interestingly, while this domain was the least represented in the discussions, parents most frequently emphasised needs which spanned across the practical, emotional, and informational domains, reflecting the day-to-day challenges and complexities of caring for a child with EB. The findings provide critical insights into the lived experiences of parents and the specific challenges they are faced with daily, highlighting significant gaps in existing supports and identifying opportunities for meaningful improvement. These include the development of initiatives that foster greater social connection and peer support among families affected by EB, and services that provide much-needed respite for parents in their caregiving roles. Practical assistance, tailored informational resources, and strategies to support emotional well-being emerged as particularly critical areas where healthcare providers, policymakers, and advocacy organisations such as DEBRA can work collaboratively to make tangible differences in the lives of families. Ultimately, this research underscores the urgent need for enhanced and coordinated support from government, healthcare systems, and patient advocacy groups to address the multifaceted challenges faced by families living with EB in Australia. Strengthening support structures and aligning resources to the identified domains of need has the potential to significantly improve the quality of life for both children with EB and their families.

## Data Availability

The datasets used and/or analysed during the current study are available from the corresponding author on reasonable request.
